# Why do older adults living alone in cities cease seeking assistance? A qualitative study in China

**DOI:** 10.1186/s12877-022-03217-x

**Published:** 2022-06-29

**Authors:** Qianqian Du, Ni Gong, Qin Hu, Guicheng Chen, Jingyue Xie, Lan Luo, Yu Cheng, Meifen Zhang

**Affiliations:** 1 School of Nursing, Sun Yat-Sen University, No.74, Zhongshan Road II, Guangzhou, Guangdong China; 2 School of Nursing, Jinan University, Guangzhou, Guangdong China; 3 School of Sociology and Anthropology, Sun Yat-Sen University, Guangzhou, Guangdong China; 4 Guangzhou Tianhe District Qizhi Social Work Service Center, Guangzhou, Guangdong China; 5 Huangpu District Hongshan Street Community Health Service Center, Guangzhou, Guangdong China; 6 The Seventh Affiliated Hospital Sun Yat-Sen University, No.628, Zhenyuan Road, Xinhu Street, Guangming New District, Shenzhen, Guangdong China

**Keywords:** Older people, Living alone, Assistance, Long-term care, Qualitative study

## Abstract

**Background:**

Against the background of an aging population, older adults living alone in cities are increasingly dependent. However, their use of home and community-based services in China is unsatisfactory. This study attempted to figure out why older adults living alone in cities do not actively seek assistance in China.

**Methods:**

In-depth interviews were conducted. A total of 29 older adults were recruited. Content analysis was used to analyze the interview data.

**Results:**

Three themes were identified. (1) Desire for independence, despite hardship: The lives of older adults involve many inconveniences, but they preferred to solve problems by themselves, instead of asking for help; (2) Desire to not overburden jiaren (meaning family in Chinese): older adults did not want to disturb families or burden children with caring responsibilities. Moreover, previous experience of failing to obtain care made them reluctant to seek help from jiaren; (3) Desire to not bother wairen (meaning people other than family in Chinese): The lack of trust caused by being unfamiliar with wairen, and the fear of being a burden to others if they were not able to reciprocate, made older adults reluctant to seek help from wairen.

**Conclusions:**

Changes in social, economic, and demographic structures have led to gradual failure of family care. Older adults accustomed to an “acquaintance society” have not yet adapted to seeking help from the community. When addressing the problem of care for older adults living alone in cities, it is important to focus on the profound impact of social change.

## Introduction

Population ageing has been given increased attention worldwide, especially in China which has the largest aging population. By the end of 2020, China’s elderly population aged 60 and above reached 264 million, accounting for 18.7% of the total population [1]. While older adults living alone tend to be dependent on others for help in daily life, as well as psychological needs, and medical care, establishing how to care for this group is a challenge globally [2–4]. Unmet needs may lead to a high incidence of accidents, psychological problems such as loneliness and “empty-nest syndrome”, and cognitive impairment, which may seriously affect quality of life [5–9]. The provision of family care and home and community-based services (HCBS) provides a potential solution for this problem. Traditional Chinese culture emphasizes the importance of family care, and proverb of “bringing up children for old age” expresses that social expectations require adult children to take care of their aging parents. However, declining family size and increasing population mobility have led to a growing number of adult children living apart from their aging parents and struggling to care for them [10]. HCBS allows older adults to live independently in the community or in their usual living environment while receiving care from the community [11, 12], such as assistance with daily tasks, personal care, meal preparation, assessment and monitoring of population health status, and other essential public health services [13–15]. But, although HCBS provides diverse and multi-level resources and help for older adults in China, the utilization of HCBS in practice is unsatisfactory. The area of care assistance with the highest uptake is home visits, but even this has only 3.9% utilization [16]. The rate of coverage of urban community hospitals and medical service institutions has reached 85.9%, but the utilization rate of each project is less than 5% [16]. In addition, the utilization of social support by older adults living alone in cities is only about 35%, which is mainly due to an unwillingness to actively seek help from family and society [17, 18]. This phenomenon raises two important questions: Why do older adults living alone in cities not seek help from their families and communities, even if they have clear care needs? and why do they refuse to accept the wealth of public care resources in their community?

Previous studies have focused on the possible reasons for underutilization of HCBS in older adults. Although older people are the main consumers of healthcare services, stereotypes, prejudice, discrimination, and structural obstacles such as inconvenient transportation, shortage of geriatric specialists, and the spread of digital healthcare make it difficult for older people to access relevant resources [19, 20]. Our research team conducted a qualitative study to reconstructed the paths and obstacles encountered by older adults in seeking community care services, and found multiple barriers, which were lack of community care information, limited mobility, complex process of achieving care, and incomprehension of needs expression, deterred the access of care resources for older adults effectively [21]. The quality of community care services may need to be improved, as service providers tend to provide care in the most convenient way, which may not be suitable for older adults [22]. As a result, medical care and nursing facilities are imperfect, spiritual and cultural provision is deficient, and it is difficult to match the complex medical care needs and personal, spiritual, and cultural pursuits of older people. In terms of community care, it is important to integrate resources within the community (i.e., family, friends, and residents within the community) to help provide care. When the social support of older adults is reduced, the utilization of services also decreases [23]. However, despite having close family or friends, older adults still seem to prefer to overcome difficulties on their own rather than seek help, in order to avoid being a burden to others [24–27]. Older adults who are at an advanced age, live alone, with financial difficulties and no medical insurance, and have lower participation in community activities are likely to have a lower utilization of social support due to lack of information and a limited range of activities [18, 19, 28, 29].

In summary, previous studies have considered the help-seeking behavior of older adults in the context of community and family, and the availability of care resources for older people has been highlighted. However, individual behavior is closely related to social background. In the current context of aging and demographic changes, it is still necessary to focus on how social change influences the use community care services among older adults [30]. Therefore, this study explores the reasons that prevent older adults living alone from seeking help, through the use of in-depth interviews with qualitative research methods.

## Methods

### Study design and sampling

In-depth interview was used in this study. This study was conducted in two urban communities in Guangzhou, South China between September 2019 and December 2020. Guangzhou is located in the south of China. This is one of the most developed cities in China, with the gross domestic product (GDP) reached 437.1 billion dollar in 2021. In 2020, adults aged 60 and above in Guangzhou reached about 1.8 million, accounting for 17% to 19% of the household registration population [31]. It is predicted that the proportion of older adults of Guangzhou will be doubled by 2030 [32]. Samples were recruited by purposive sampling. In China, “older adults” are defined as ≧60 years [33]. Our subjects were required to meet the following inclusion criteria: (1) 60 years of age or older, (2) living alone, (3) able to speak Mandarin or Cantonese, and (4) willing to participate in this study. Data were collected until saturation of information was achieved. In this study, the data reached saturation when interviewing the 26^th^ older adult. After 3 further interviews were conducted with no new themes emerging, recruitment was stopped. Overall, 29 older adults living alone in cities completed the interviews (Table 1). The duration of the interviews ranged from 25 to 77 min, with an average time of 50.69 ± 15.36 min.

**Table 1:** Description of older adults in the study

No	Gender	Age	Physical condition	History of professional occupation
P2	Female	60	Respiratory disease, osteoporosis, fracture (cane required for walking)	Staff of a public institution
P3	Female	85	Fracture, hypertension, loin and leg pain, poor sleep quality	Professor
P4	Female	83	Terminal cancer	Individual business
P5	Female	83	Arteriosclerosis, fracture, hip replacement, poor sleep quality	Staff of a research institute
P6	Female	83	Poor sleep quality, frequent colds	Staff of state-owned enterprise
P7	Female	88	Heart disease, atherosclerosis, osteoporosis, knee pain	Staff of civil service
P8	Female	83	Coronary heart disease, facial neuritis, knee arthritis, cervical spondylitis, scapulohumeral periarthritis	Staff of state-owned enterprise
P9	Female	81	Arthritis, limb pain, indigestion, bone tuberculosis, poor sleep quality	Professor
P10	Female	83	Hypertension, diabetes, arthritis	Staff of a public institution
P11	Male	74	Hypertension	Worker
P12	Male	75	Hypertension, arthritis	Staff of a public institution
P13	Female	83	Arthritis, poor sleep quality	Staff of state-owned enterprise
P14	Female	85	Fracture, poor vision, hypertension	Staff of a research institute
P15	Male	83	Hypertension, Parkinsonism	Staff of a college
P16	Male	80	Hypertension	Staff of a public institution
P17	Male	87	Lung cancer	Staff of a public institution
P18	Female	81	Hypertension, arthritis	Staff of a public institution
P19	Male	81	Hypertension, hyperlipidemia	Individual business
P20	Female	86	Hypertension, heart disease, diabetes	Staff of a research institute
P21	Male	85	Arthritis; cane is needed while walking	Individual business
P22	Female	79	Meniere’s disease, osteoporosis, vegetative system dysfunction, irritable bowel syndrome	Staff of a research institute
P23	Female	82	Heart disease, hypertension, osteoporosis	Staff of a public institution
P24	Female	69	Gout	Worker
P25	Female	81	Hypertension, diabetes, respiratory disease, osteoporosis	Business personnel
P26	Male	81	Hypertension, liver cirrhosis	Individual business
P27	Male	83	Hypertension, stroke, nerve deafness	Teacher
P28	Female	76	Heart disease, hypertension	Worker
P29	Female	72	Heart disease, respiratory disease, arthritis, osteoporosis, depression	Worker

### Procedures

This study relied on social workstations, health service centers, neighborhood committees, and other community organizations. Older adults were recruited when they participated in community activities organized by the above organizations, or as a member of the case consultation. The interviews were conducted in a separate meeting room or in the homes of older adults living alone in cities to guarantee a quiet and private environment. QD and QH conducted semi-structured interviews with older adults living alone.

An initial interview guide was developed on the basis of a literature review [34]. After being revised by a gerontologic nursing specialist, a nursing anthropology specialist, and a social worker, the following questions were included in the final interview guide: (1) What is your daily life like? (2) What difficulties do you usually encounter in your daily life? (3) How do you solve them when facing those difficulties? (4) Why don’t you try to ask others for help? Detailed questions were asked at appropriate times to obtain further details.

### Data analysis

Content analysis was used to analyze the interview data. The interview materials were transcribed within 24 h after interview, and then encoded by QD and QH, respectively. Discrepancies were discussed by the research team (including a geriatric nursing professor, medical anthropologists, social workers in the field of gerontology, and nursing students) until consensus on how to sort the codes was reached. According to the requirements of the content analysis [35], each researcher ensured they were familiar with the interview materials and had a good understanding of the overall situation. Then, the interview materials were analyzed sentence-by-sentence, and the contents related to the research were coded and integrated to form a theme. Next, links between the themes were identified in order to finish the thematic framework. The above steps were repeated until no more topics were generated and information saturation was reached.

## Results

Lack of assistance for older adults living alone in cities could put them at risk. However, despite these difficulties, many older adults believe that they are able to live alone and can cope with the potential risks. The data from this study identified three themes (Table 2) to describe why older adults living alone in cities do not actively seek help from others: (1) desire for independence, despite hardship; (2) desire to not overburden jiaren; (3) desire to not bother wairen.

**Table 2:** Three themes and related subthemes

Theme	Subthemes
1. Desire for independence, despite hardship	1. Difficulties associated with living alone
	2. Not asking for help, despite the inconvenience of living alone
	3. Working out how to solve problems in order to avoid the risks of living alone
2. Desire to not overburden jiaren	1. Not wanting to disturb jiaren
	2. Not wanting to burden children with caring stress
	3. “No expectation, no disappointment”
3. Desire to not bother wairen	1. Not familiar enough with wairen to ask for help
	2. Insufficient trust in wairen to ask for help
	3. Concerns about not being able to pay wairen for help
	4. Not wanting to waste resources and feeling embarrassed to ask for help

### Desire for independence, despite hardship

#### Difficulties associated with living alone

Some older adults living alone in cities were no longer able to cope with simple daily activities, such as housework, shopping, and cooking. From their descriptions of daily life, it was obvious that such older person had encountered a variety of inconveniences in their life.

For example, an elderly woman who lived alone had been suffering from leg problems since she fell over when trying to replace a bucket 4 years ago. However, she still insisted on cleaning the room by herself:

I usually do everything on my own, such as cleaning my house. I was a farmer, and I’m not afraid of being tired. I can do anything... (P1)

Similarly, another older person had a hip and knee replacement 3 years ago because of a fall, and now needed to walk with crutches and worried about falling again; she still insisted on living alone. When she was asked how she coped with daily life with limited mobility, she said:

I live alone, and I do all the shopping and cooking things by myself... I don’t want to rely on others... I have to do it on my own. (P5)

One of the risks faced by older adults who live alone is being left undetected and unattended if they become ill, potentially endangering their life. The clinical manifestations of Meniere's disease are recurring episodes of vertigo, hearing loss. ringing in the ear, and feeling of fullness in the ear. One respondent with Meniere’s disease had repeated episodes over recent years, describing a particularly frightening incident in February 2021:

I was cooking at noon. Suddenly, I felt dizzy and fell on the ground while still conscious. Because both gas stoves were on, I was frightened and thought it could be dangerous. I had to reach the gas stoves with all my strength and turn them off before danger occurred. (P22)

#### Not asking for help, despite the inconvenience of living alone

Despite the problems and potential risks, older adults living alone in cities continued to insist on looking after themselves.

Because I have Meniere’s disease, my family thinks it’s dangerous for me to live alone... My daughter tries to convince me to have a housekeeper, but I refuse. I’m not at the point where I need someone to take care of me. As long as my illness doesn’t get worse, I can take good care of myself. (P22)

To be honest, there are a lot of inconvenient things in my solitary life... I have few needs, and I’ll make do with my life... I do everything mainly on my own. (P27)

#### Working out how to solve problems in order to avoid the risks of living alone

The older adults living alone in cities insisted that they should find ways to solve the problem by themselves, even though they lived with difficulties and risks every day. Instead of actively seeking help from family members or community care workers, they had come up with “clever” ideas.

The participant with Meniere’s disease described above had repeatedly talked about her worries about being left alone and unattended in the case of illness. Over time, she developed her own set of solutions:

I always get on a random bus, go to the terminal, and then go back. I do that over and over again... As long as there are people around me, I’m not afraid. Because wherever there are people, there is help... Sitting on the bus makes me have a sense of security. (P22)

One participant who had been living with liver cirrhosis for 12 years lives in the same city as his son, who is a professor at a university medical school and able to provide his father with medical advice. However, he spent 2 months teaching himself how to register at the hospital with a smart-phone, and did not ask his son to register or find a doctor for him.

I have to learn to register by smartphone! There is no alternative. This is progress – otherwise I will be left behind. (P26)

### Desire to not overburden jiaren

Jiaren means a family member in Chinese. Familism and collectivism are emphasized in Chinese tradition, such that adult children and other family members jointly provide care for older people. In this context, it is taken for granted that older adults should receive necessary daily care and emotional support from families. However, there were many reasons why older adults faced obstacles in asking families for help.

#### Not wanting to disturb jiaren

Jiaren refers to people who are related by blood, and includes the children, brothers, and sisters of older people. Although family care has traditionally been the predominant care model for older adults in China, our interviews found that older adults living alone in cities tended to be independent of the “small family” of jiaren, and considered seeking help from them was undoubtedly a disturbance to them.

Why don’t I go live with my little brother? He has his family. It would be disruptive for him to do that. (P24)

My kids suggested that I should live with them, but I refused them! I might as well live in my own house... Anyway, this is my home. Why would I go to someone else’s home? (P23)

#### Not wanting to burden children with caring stress

Traditional Chinese culture focuses on filial piety and emphasizes the role of older adults in the family. Therefore, children caring for older adults has always been the most important model of care in China [36]. However, our study found that older adults made allowances for the pressure on their children, and preferred the inconvenience of living alone to putting extra stress on their children.

My son lives in the same city as me, but he’s always on business trips and as busy as a bee... So even if something happened, I would barely ask him for help or bother him. I can do things on my own, and I don’t want to rely on others. (P20)

My daughter has a grandson born recently, which makes her very busy every day... She needs to pay more attention to her son and grandchild... I always tell her she needn't come to see me. (P14)

#### “No expectation, no disappointment”

For other older adults, expectations of receiving care from their children were not realized, leading to disappointment. To avoid repeating this psychological setback, some older adults living alone in cities no longer actively asked their children for help.

My daughter is very busy. There was a time when I wanted to chat with her to divert myself from boredom, so I went to her workplace to find her. But she scolded me and said, “Don’t bother me when I’m working!” After that, I’m unwilling to ask my daughter for help. (P4)

They (my son and my daughter) cannot help me much! “Bring up sons to support parents in their old age” is empty talk! What if I couldn’t take care of myself? We’ll see when we get there... (P27)

### Desire to not bother wairen

In addition to jiaren, older adults might turn to wairen for support when they encountered difficulties. Wairen means people other than family members in Chinese, including but not limited to neighbors, friends, and members of voluntary organizations and public authorities. For older adults, informal care provided by neighborhoods and communities was the easiest way to reach out to wairen. However, in modern cities, interpersonal relationships have changed, creating obstacles for older adults to get care and help from their neighbors and communities.

#### Not familiar enough with wairen to ask for help

The old Chinese saying, “A good neighbor is better than a brother far off”, illustrates the trust and reliance placed on neighbors. However, mass migration of populations and changes in urban living mean that older adults were no longer surrounded by familiar neighbors, but by unknown renters or new homeowners.

The people living around here are mostly unknown renters or new homeowners. I barely see them or say hello. Naturally, I have never thought about asking for their help. (P17)

My range of social activities has gradually narrowed day by day. I am not familiar with the people around me, which makes me embarrassed to bother them with my own affairs. (P10)

#### Insufficient trust in wairen to ask for help

Informal care provided by communities was one of the most significant ways that older adults living alone in cities could obtain assistance. In order to provide appropriate services, community social workers usually needed to know their personal information, or make a house visit, which made older adults reluctant to seek help from ones without a trusting relationship.

When I wanted help with housecleaning, I had to fill out an application form, which required information about my telephone number, address, how many children I have, and even where my children live... I’m too old to be defrauded... I resent people knowing too much about me... After that, I’d rather do the hard work of cleaning up than seek help from them again. (P18)

I’ve received calls from the community saying they want to come home and talk to me and find out what they can do to help me, but I refuse... Old people like me, who live alone, are afraid to let strangers into our homes. (P29)

#### Concerns about not being able to pay wairen for help

The above findings showed that older adults living alone in cities found it difficult to ask for help from neighbors who were not familiar. However, even in the case of friendly neighbors who were willing to help, older adults might be afraid that they were not able to reciprocate, so would still not seek help from neighbors. As one participant said:

My neighbor at the market downstairs of my house knows I am old. She suggested that she could help deliver vegetables and meet to me upstairs... But I know that an old woman with an limited ability to walk like me can’t offer assistance to anyone. (P2)

Actually, my neighbors are enthusiastic and willing to help me. However, if I accept their kindness, I’ll owe them; so it’s better not to bother them. (P19)

#### Not wanting to waste resources and feeling embarrassed to ask for help

Theme 3.2.1 from this research (Not wanting to disturb jiaren) showed that older adults living alone in cities did not want to disturb their families with their problems. They also seemed to have the same attitude toward care providers and other resources. Although some older adults were entitled to benefit from these sources of help, they did not take the initiative to apply because they did not want to waste public resources or become a burden to others.

An 83-year-old elderly was entitled to seek help from caregivers for cleaning and accompanying to visit the doctor, but she almost never made use of these resources:

I’m getting old, and I know it is the law of nature... There must be people in worse health than me. If they can move on, why can’t I? I don’t want to waste any resources. (P10)

Similarly, a 74-year-old man also never applied for these resources:

I have free medical service and a higher retirement pay than others. I could be a trouble-maker if I’m not satisfied with this, but make unreasonable demands. (P11)

The community can provide meals for older people, and volunteers can deliver them to their homes. But one elderly man, who has walked on crutches for years, did not apply. When asked why, he said:

I buy food and cook by myself. I do it if I can. I don’t want to annoy others. It’s too much trouble for them to deliver food. (P21)

## Discussion

Difficulties faced by older adults living alone in cities include declining health status, being left unattended, loneliness, and the risk of not being able to get timely medical treatment. This study found that despite these difficulties and risks, many older adults had to cease seeking assistance. Combining the backgrounds of demographic structure and social interpersonal relationship in China, we tried to explain the reasons why older adults living alone in cities did not seek help from both jiaren and wairen.

Aging means not only a physical decline, but also an increase in dependence on social support, especially for those living alone [37, 38]. Previous studies have highlighted that although older adults may regard accepting help from others as a stigma, older age can be a legitimate excuse for accepting help when people are no longer able to manage on their own [34, 39]. This is why many countries are emphasizing the importance of the community in dealing with the care needs of older adults living alone in cities [40]. However, China’s care system of older adults in particular is in a period of great transformation due to the influence of social, economic, demographic, and other structural factors. The traditional model of providing for older adults through the support of families and adult children is experiencing unprecedented challenges. Sub-replacement fertility levels and the atomization of social life make it more difficult for children to care for older adults, while it is difficult for older adults to accept the emerging community care system due to the short development period and immaturity of the scheme. To a large extent, this phenomenon has resulted in the difficult situation in which older adults turn neither to jiaren nor to wairen for help. Therefore, there is a practical importance in exploring the reasons behind this seemingly abnormal behavior from the perspective of social culture.

Family is regarded as the basic unit of social relations in China. With filial piety as its core, traditional Chinese culture also emphasizes the role of older adults in the family, which makes family care the ideal choice for older adults [41, 42]. However, in just 40 years, China’s “one child policy” has led to “inverted pyramid” structure for many families [43]. This inevitably means that the middle-aged breadwinners, especially those who live in metropolis, must take on not only the pressure of providing care for older family members, but the increasing burden of raising children [44, 45]. In addition, descending familism, in which a family’s resources are directed toward the children and grandchildren, has become a common phenomenon. Thus, the children of older adults living alone in cities are required not only to manage their own work and life, but provide downward intergenerational support and intergenerational parenting [46–48]; this is bound to undermine to some extent their role as a pillar of care for older adults. Not wanting to be a burden to their children, older adults are therefore forced to accept the fact that they cannot rely on the younger generation, and have no choice but to manage the inconveniences and difficulties of life on their own [27]. Some older adults who were ignored or rejected after seeking care from their children may become sensitive and avoid further communication, no longer taking the initiative to ask for help [49]. The combined effects of the above factors mean that the role of family care has been impacted in an unprecedented way, limiting the availability of care from families or children, and reducing the chance of older people asking for help.

When the traditional family care system of older adults is challenged due to the above reasons, the rational choice for older adults living alone in cities, especially for the participants in this study who live in Guangzhou — one of the most developed cities in China and with more well-established home and community-based services, should be to seek help from wairen [50]. However, this is still difficult for older adults who usually have conservative and traditional values. After more than 40 years of reform and opening-up, China has made great socio-economic achievements. However, it has also made cities be more away from the concept of the “acquaintance society”, as proposed by Chinese sociologist and anthropologist, Fei Xiaotong, which refers to a network of relationships through which people are connected [51]. Specifically, for older adults living alone in cities, this concept describes the alienation of neighbors and the lack of familiarity, understanding, and use of new social resources. Carstensen's socioemotional selectivity theory also supports this point of view. According to this theory, future time perspective (refers to one’s perception of time and of how much time is left) affects the composition of their social network. Specifically for older adults, they may terminate peripheral social relationships to focus on those that are particularly important because of a substantial reduction in the size of their social network. As a result, while they can not get help from jiaren, it is also difficult for them to ask for help from those unfamiliar wairen [52, 53]. In addition, social participation among older adults with chronic diseases declines, making it difficult to maintain long-standing social circles [54]. Furthermore, the sense of self-worth of older adults, especially those living alone, has declined [55, 56]. As a result, older adults may feel unable to reciprocate when offered help, worry about being a burden or being mocked, and are afraid to inconvenience others. Because of this, it is difficult for older adults living alone in cities to ask for help.

### Limitations

This study is not without limitations. Firstly, the study was conducted in an economically developed city, where the older adults are better off financially. However, considering the the burden of chronic disease and the inconvenience of living alone, it's still worth a larger sample to explore why they do not seek help. Future research involving older adults in less developed cities and rural areas is needed to investigate the variation of this issue. Secondly, the research questions related to family issues, which might be not suitable to be talked about in public in China, socially desirable answers could not be fully excluded and may have influenced their answers.

## Conclusion

It is clear from this study that there may be tension between the fragility and dependence of older adults living alone in cities and the independence they are trying to demonstrate, which indirectly prevents them from recognizing their need for self-care. The gradual failure of familiar and accustomed family care makes older adults forced to seek help from strangers. However, the alienation of neighbors and the lack of understanding of new social resources make it inappropriate to ask for help from them. When addressing the problem of care for older adults living alone in cities, it is important to focus on the profound impact of social change, and to establish a community care model that is more accessible and comprehensible, so that older adults living alone in cities who are confused by the social transformation in the process of urbanization can be better cared for.

## Data Availability

The datasets generated and analyzed during the current study are not publicly available in order to maintain participant privacy and confidentiality but are available from the corresponding author on reasonable request.
